# Ischemic post-conditioning in acute ischemic stroke thrombectomy: A phase-I duration escalation study

**DOI:** 10.3389/fnins.2022.1054823

**Published:** 2022-12-08

**Authors:** Longfei Wu, Bohao Zhang, Wenbo Zhao, Xunming Ji, Ming Wei

**Affiliations:** ^1^Department of Neurology, Xuanwu Hospital, Capital Medical University, Beijing, China; ^2^Department of Neurology, Tianjin Huanhu Hospital, Tianjin, China; ^3^Department of Neurosurgery, Xuanwu Hospital, Capital Medical University, Beijing, China; ^4^Beijing Institute for Brain Disorders, Capital Medical University, Beijing, China; ^5^Department of Neurosurgery, Tianjin Huanhu Hospital, Tianjin, China; ^6^Tianjin University, Tianjin, China

**Keywords:** acute ischemic stroke, mechanical thrombectomy, ischemia-reperfusion injury, ischemic post-conditioning, dose-escalation

## Abstract

**Background:**

Previous experimental studies have found that ischemic post-conditioning exhibits neuroprotective effects by alleviating ischemia-reperfusion injury in an acute ischemic stroke model, and its efficacy is thought to be related to the duration of ischemic post-conditioning. However, ischemic post-conditioning has not been used in patients with acute ischemic stroke. This study aims to determine the safety, tolerability, and maximum tolerable duration of ischemic post-conditioning in patients with acute ischemic stroke receiving mechanical thrombectomy.

**Methods:**

Patients with acute ischemic stroke with unilateral middle cerebral artery M1 segment occlusion eligible for mechanical thrombectomy will be enrolled. We adopt a 3 + 3 dose-escalation design with a duration escalation schedule of 0, 1, 2, 3, 4, and 5 min × 4 cycles for the ischemic post-conditioning study. After successful reperfusion following mechanical thrombectomy, the balloon for ischemic post-conditioning will be inflated at the site proximal to the culprit lesion four times for 0–5 min with low-pressure (3–4 atmospheres) inflations, each separated by 0–5 min of reflow. We pre-defined the major responses (vessel perforation or rupture, reocclusion of the culprit vessel after ischemic post-conditioning, vessel dissection, severe vasospasm, ischemic post-conditioning related thrombotic events, and rupture of the balloon used for ischemic post-conditioning) as the stopping rules. Each patient will undergo a rigorous evaluation to determine the safety, tolerability, and maximum tolerable duration of ischemic post-conditioning.

**Discussion:**

This will be the first clinical study to ascertain the safety and tolerability of ischemic post-conditioning in patients with acute ischemic stroke receiving mechanical thrombectomy. The maximum tolerable duration obtained in this study will also serve as a starting point for future studies on the efficacy of ischemic post-conditioning.

**Clinical trial registration:**

[https://clinicaltrials.gov], identifier [NCT05153655].

## Introduction

Acute ischemic stroke, the most common type of stroke, is caused by decreased cerebral blood flow due to occlusion of cerebral arteries (Campbell and Khatri, 2020). Recanalizing the occluded vessels and restoring the reperfusion of the tissue at risk are the key strategies for the treatment of acute ischemic stroke. Mechanical thrombectomy is a standard recanalization therapy and has been recommended by guidelines after multiple randomized clinical trials have demonstrated that mechanical thrombectomy improved patient outcomes compared to standard medical therapy alone (Powers et al., 2018). However, even after mechanical thrombectomy, less than 50% of patients are functionally independent at 90 days, and mortality exceeds 15% (Goyal et al., 2016). Previous studies have focused on the role of inflammation in the pathogenesis of ischemic stroke and its relationship with reperfusion mechanisms (Pinto et al., 2006; Tuttolomondo et al., 2015, 2016). Recently, ischemia-reperfusion injury after recanalization is thought to play a prominent role in the poor prognosis of patients undergoing mechanical thrombectomy, as abrupt reperfusion induces secondary brain damages (De Meyer et al., 2016; Sutherland et al., 2016; Zhao et al., 2020). Therefore, alleviating ischemia-reperfusion injury after recanalization may further improve the functional outcomes of patients with ischemic stroke (Wills and Ding, 2020).

Ischemic post-conditioning is a promising therapeutic strategy in which several cycles of ischemia and reperfusion performed immediately after reflow confer protection against more severe injuries (Zhao et al., 2003). This strategy has been widely investigated in coronary heart disease. A randomized clinical trial indicated that ischemic post-conditioning reduced infarct size and edema caused by ischemia-reperfusion injury in patients with reperfused ST-segment elevation myocardial infarction (Thuny et al., 2012). A number of experimental studies using rodent animal models also demonstrated that ischemic post-conditioning reduced infarct volume and improved neurological outcomes, thereby inducing neuroprotection after stroke (Zhao et al., 2006; Esposito et al., 2015; Doeppner et al., 2017). However, whether ischemic post-conditioning has neuroprotective effects in patients with acute ischemic stroke undergoing mechanical thrombectomy remains unknown.

In this study, we plan to conduct a 3 + 3 dose-escalation trial to determine the safety and tolerability of ischemic post-conditioning incrementally for a longer duration of up to 5 min × 4 cycles in stroke patients undergoing mechanical thrombectomy. The present study, called the Ischemic Post- conditioning in Acute Ischemic Stroke Thrombectomy (PROTECT) study, will also provide the basis for a subsequent prospective study to explore the efficacy of ischemic post- conditioning.

## Methods

This is a single-center, phase-I, dose-escalation study to investigate the safety and tolerability of ischemic post-conditioning in stroke patients undergoing mechanical thrombectomy. This study was approved by the Local Ethics Committee of Tianjin Huanhu Hospital and registered at Clinicaltrials.gov (Identifier: NCT05153655; Registration date: 10 December 2021^1^). All patients will voluntarily sign an informed consent form before being enrolled in this study.

### Study design

**FIGURE 1:**
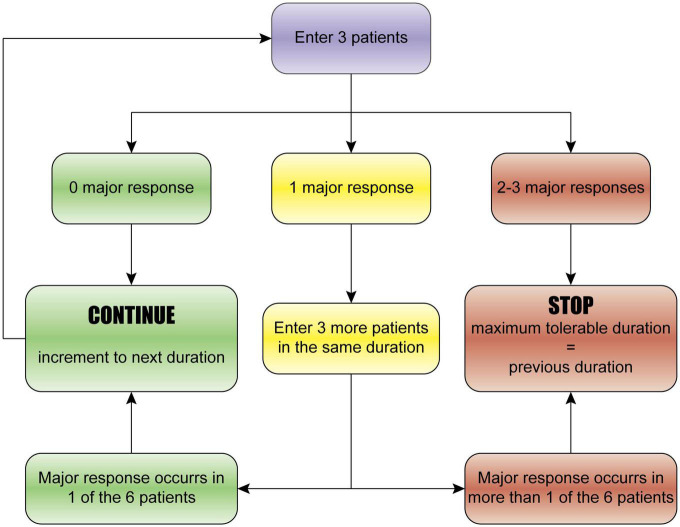
The 3 + 3 dose-escalation trial design. The study starts with three patients. (a) If no major response occurs in any of the three patients, the next duration will be used. (b) If a major response occurs in one patient, then three more patients will be enrolled to further assess the safety and tolerability of the same duration; if no additional major response occurs, the duration will be escalated to the next dose; if an additional major response occurs, the study will be stopped, and the previous dose will be defined as the maximum tolerable duration of ischemic post-conditioning. (c) If a major response happens in 2–3 patients, the study will be stopped, and the previous dose will be defined as the maximum tolerable duration. The incremental schedule of the duration is 0, 1, 2, 3, 4, and 5 min × 4 cycles.

The study protocol is designed with reference to the classical 3 + 3 drug trial (Hansen et al., 2014), which is generally used to determine the maximum tolerable dose of a drug in phase-I clinical study. The study design is illustrated in Figure 1. Specifically, the study starts with three patients in the 0 min group (sham operation). (a) If no major response (defined below) occurs in any of the three patients, 1 min × 4 cycles (1 min ischemia followed by 1 min reperfusion, and repeated for 4 cycles, total time 7 min) will be used. (b) If a major response occurs in one patient, then three more patients will be enrolled to further assess the safety and tolerability of the same duration (1 min × 4 cycles); if no additional major response occurs, the duration will be escalated to the next dose (2 min × 4 cycles; 2 min ischemia followed by 2 min reperfusion, and repeated for 4 cycles, total time 14 min); if an additional major response occurs, the study will be stopped, and the previous dose will be defined as the maximum tolerable duration of ischemic post-conditioning. (c) If a major response happens in 2–3 patients, the study will be stopped, and the previous dose will be defined as the maximum tolerable duration. The incremental schedule of the ischemia and reperfusion duration is 0, 1, 2, 3, 4, and 5 min × 4 cycles.

### Major response

We pre-defined the major responses before the start of the study, which included any of the following adverse events during the procedure:

• Vessel perforation or rupture (angiography shows active contrast extravasation from the site of ischemic post-conditioning, and postprocedural head computerized tomography shows a high-density region in the corresponding area).
• Reocclusion of the culprit vessel after ischemic post-conditioning.
• Vessel dissection (angiography shows the typical appearance of dissection at the site of ischemic post-conditioning, such as intimal flap, double lumen, pseudoaneurysm, and long tapering stenosis).
• Severe vasospasm requiring treatment.
• Ischemic post-conditioning related thrombotic events (angiography shows *in situ* thrombosis or distal embolism after ischemia post-conditioning).
• Rupture of the balloon used for ischemic post- conditioning.

### Inclusion and exclusion criteria

We will recruit patients with acute ischemic stroke who are undergoing mechanical thrombectomy. The inclusion criteria include: (1) age 18–80 years; (2) acute ischemic stroke caused by unilateral middle cerebral artery M1 segment occlusion and eligible for mechanical thrombectomy; (3) successful reperfusion after mechanical thrombectomy confirmed by digital subtraction angiography; and (4) written informed consent provided by patients or their legal relatives.

Exclusion criteria are as follows: (1) known diagnosis or clinical suspicion of cerebral vasculitis or fibromuscular dysplasia; (2) difficulty in reaching the designated position of the balloon used for ischemic post-conditioning; (3) stenting in the middle cerebral artery as rescue therapy during the thrombectomy procedure; (4) >2 balloon dilations as rescue therapy due to angioplasty during the thrombectomy procedure; (5) remnant moderate or severe residual stenosis (≥50%) of the culprit artery after thrombectomy; and (6) difficulty complying with ischemic post-conditioning or other conditions considered inappropriate for inclusion by the investigator.

### Procedures

Patients with acute ischemic stroke caused by large vessel occlusion will undergo mechanical thrombectomy, as recommended by the guidelines (Powers et al., 2018). Intravenous thrombolysis will be permitted in patients within 4.5 h of stroke onset. To reduce procedural risks, all endovascular therapies will be performed by neuro-interventionalists experienced in neurovascular interventions. The anesthesia care team, neuro-interventionalist, and neurologist will decide on the anesthetic approach (local anesthesia, conscious sedation, or general anesthesia) during the procedure in an interdisciplinary fashion, according to the level of patient cooperation and physical status.

All patients will undergo diagnostic digital subtraction angiography to determine the occluded vessels. Then, mechanical thrombectomy will be performed, with the first-line approach consisting of thrombectomy with a stent retriever or thrombo-aspiration devices. When the first-line approach to mechanical thrombectomy fails to achieve successful reperfusion, stenting, balloon angioplasty, and intra-arterial thrombolysis will be used as rescue therapies. The specific interventional strategies and devices used during thrombectomy will be chosen at the discretion of the treating neuro-interventionalist based on clinical conditions and imaging results. At the end of the procedure, reperfusion status will be assessed using the modified thrombolysis in cerebral infarction (mTICI) perfusion score (Zaidat et al., 2013). The mTICI 2b/3 is defined as successful reperfusion.

For patients enrolled in the present study, ischemic post- conditioning will begin within 1 min of reflow after successful reperfusion. Specifically, the balloon for ischemic post-conditioning (Neuro LPS, SINOMED, Tianjin, China) will be reinflated four times for 0–5 min with low-pressure (3–4 atmospheres) inflations, each separated by 0–5 min of reflow. Reinflation will be performed at the site proximal to the culprit lesion. Angiography will then be performed again to assess cerebrovascular condition.

After all procedures, patients will be transferred to the neuro-intensive care unit or the stroke unit for further treatment.

### Sample size

Since this is a 3 + 3 dose-escalation study with six doses (0, 1, 2, 3, 4, and 5 min), a minimum of 18 (6 groups × 3 patient/group) patients will be required, assuming no major response occurs at any dose level, and a maximum of 36 (6 groups × 6 patient/group) patients will be required, assuming one major response occurs at each dose level.

### Data Safety Monitoring Board

The PROTECT study will establish an independent Data Safety Monitoring Board to evaluate all the safety profiles. In addition to the above-mentioned major responses, any serious adverse events occurring during hospitalization, whether or not related to ischemic post-conditioning, must be reported to the Institutional Review Board within 24 h of their occurrence, pending review by the Data Safety Monitoring Board after investigators provide relevant data.

### Statistical analysis plan

Since this is a 3 + 3 dose-escalation study to explore safety and tolerability, the results will be described objectively. If required for statistical analysis, continuous variables will be presented as mean (standard deviation, SD) or median (interquartile range, IQR) and tested using Student’s *t*-test or Mann–Whitney U test, as appropriate. Categorical variables will be presented as absolute numbers and percentages and tested using Chi-square test or Fisher’s exact test. Throughout the analysis, the significance level will be 5% two-sided. Statistical analysis will be performed using SPSS Statistics 26 (IBM Corp., Armonk, NY, USA).

## Discussion

Although mechanical thrombectomy has significantly improved the prognosis of patients with acute ischemic stroke caused by large vessel occlusion, active intervention against ischemia-reperfusion injury post thrombectomy is still considered a promising strategy that may further improve patient outcomes (Mizuma et al., 2018). Ischemic post-conditioning, a non-pharmacological treatment that attenuates ischemia-reperfusion injury by intermittent reocclusion of the artery that perfuses the ischemic vascular bed, is effective in brain experiments.

The research on ischemic post-conditioning in the field of stroke began in 2006, when Zhao et al. (2006) demonstrated for the first time that ischemic post-conditioning with a series of interruption of reperfusion reduced infarct size after focal stroke as a function of stroke severity. Esposito et al. (2015) also found that ischemic post-conditioning significantly reduced infarction and improved neurological outcomes in stroke rats. Doeppner et al. (2017) again provided evidence that ischemic post-conditioning induced neuroprotection. They found that post-conditioning reduced infarct volume and neurological deficits 24 h post-stroke, enhanced blood-brain barrier integrity, reduced brain leukocyte infiltration, and reduced oxidative stress, which was likely to be meditated *via* Hsp70-induced proteasome inhibition and NF-κB deactivation. Meanwhile, other researchers have successively discovered the neuroprotection of post-conditioning against stroke in their own laboratories (Pignataro et al., 2008; Taskapilioglu et al., 2009; Li et al., 2012; Han et al., 2014; Liang et al., 2014). However, owing to the immaturity of recanalization therapy in the field of stroke over the past few decades, it has been difficult to fully evaluate the effects of ischemic post-conditioning on ischemia-reperfusion injury in stroke patients through clinical studies. With the increasing popularity of mechanical thrombectomy, it is now time to evaluate the role of ischemic post-conditioning in stroke patients. As a matter of course, it is necessary to test safety and tolerability before efficacy can be verified.

We designed the PROTECT study based on the traditional 3 + 3 dose-escalation trial, which is widely acknowledged as a standard method for determining the safety, tolerability, and maximum tolerable dose of a treatment modality (Chhatbar et al., 2017). The incremental design concept of the 3 + 3 dose-escalation trial is also consistent with our original intention to explore the optimum effective duration of ischemic post-conditioning that is safe and tolerable for stroke patients. One may be concerned about the potential unfavorable effect of transient ischemia. In the present study, the maximum duration of a single ischemia was 5 min, which has not yet reached the time leading to neuronal death. Additionally, the ischemic brain tissue can still receive blood supply from collateral circulation even when the vessel is reoccluded by the balloon. We will include stroke patients who achieved successful reperfusion after mechanical thrombectomy, meaning that the time from stroke onset to reperfusion is mostly several hours. Compared with several hours to achieve successful reperfusion, transient ischemia of 0–5 min does not appear to have a significant unfavorable effect, let alone transient ischemia followed by reperfusion. On the other hand, the duration of ischemic post-conditioning has varied across studies, but a longer duration is likely more efficacious in alleviating ischemia-reperfusion injury and enhancing neuroprotection (Pignataro et al., 2008).

## Conclusion

In conclusion, the PROTECT study aims to assess the safety and tolerability of ischemic post-conditioning in patients with acute ischemic stroke receiving mechanical thrombectomy. The maximum tolerable duration obtained in this study will also serve as a starting point for future studies on the efficacy of ischemic post-conditioning. Upon completion of the study, the results will be disseminated at academic conferences and submitted to a relevant peer-reviewed journal for publication.

## Ethics statement

The studies involving human participants were reviewed and approved by the Local Ethics Committee of Tianjin Huanhu Hospital. The patients/participants provided their written informed consent to participate in this study.

## Author contributions

LW, WZ, XJ, and MW conceptualized and designed the study. LW, BZ, and WZ contributed to the study design and content. LW, BZ, and WZ were involved in the design, implementation, and wrote the manuscript. All the authors approved the final version of the manuscript.

## Footnotes

1
clinicaltrials.gov/ct2/show/NCT05153655
